# Immunization Using GroEL Decreases *Clostridium difficile* Intestinal Colonization

**DOI:** 10.1371/journal.pone.0081112

**Published:** 2013-11-26

**Authors:** Séverine Péchiné, Claire Hennequin, Céline Boursier, Sandra Hoys, Anne Collignon

**Affiliations:** 1 Université Paris-Sud, Faculté de Pharmacie, Equipe d’Accueil 4043, Unité Sous Contrat Institut National de la Recherche Agronomique, Châtenay-Malabry, France; 2 Clermont Université, Unité Mixte de Recherche Centre National de la Recherche Scientifique, Laboratoire Microorganismes: Génome Environnement, Université d’Auvergne, Clermont-Ferrand, France; 3 Université Paris-Sud, Faculté de Pharmacie, plate-forme TransProt, Institut Paris-Sud d'innovation thérapeutique, Châtenay-Malabry, France; Institute Pasteur, France

## Abstract

*Clostridium difficile* is a pathogen which is responsible for diarrhea and colitis, particularly after treatment with antibiotics. Clinical signs are mainly due to two toxins, TcdA and TcdB. However, the first step of pathogenesis is the colonization process. We evaluated *C. difficile* surface proteins as vaccine antigens in the hamster model to prevent intestinal colonization. This vaccination induced a partial protection of hamsters against death after a *C. difficile* challenge. A proteomic analysis of animal sera allowed us to identify proteins which could be responsible for the protection observed. Among these proteins, we identified the GroEL heat shock protein. To confirm the role of the specific GroEL antibodies in the delayed *C. difficile* colonization of hamsters, we performed an immunization assay in a mouse model. After intranasal immunization with the recombinant protein GroEL, we observed a lower *C. difficile* intestinal colonization in the immunized group as compared to the control group.

## Introduction

Following disruption of intestinal microbiota by antibiotics, *C. difficile* colonizes the intestinal tract, resulting in a spectrum of disease from asymptomatic carriage to pseudomembranous colitis (PMC) [1], [2], [3]. The disease symptoms are mediated by two enterotoxins TcdA and TcdB. *C. difficile* is shed in feces as vegetative cells and spores that persist in the environment and facilitate cross-contamination and relapses [4].

After colonization by *C. difficile*, the host immune response is considered of prime importance in preventing disease. In fact, an immune response to TcdA, during an initial episode of *Clostridium difficile* infection (CDI), has been associated with protection against recurrences [5]. A vaccine based on formaldehyde-inactivated TcdA and TcdB has been developed and used in healthy volunteers, and induced high levels of specific neutralizing IgG. Initial studies have been conducted with promising results in a few patients with recurrent CDI [6].

Although the role of anti-toxin immunity in protection against CDI is clear, vaccines based on toxins are unlikely to prevent colonization. The carriage and transmission of *C. difficile* therefore remain a persistent threat. A more complete approach against CDI should consider not only the inhibition of toxicity, but also the prevention of bacterial colonization.

To date, the colonization mechanism remains to be elucidated [7]. Proteomic analysis of cell surface proteins of *C. difficile* led to the discovery of a number of adhesion factors suggesting that there may be a whole consortium of proteins involved in the attachment of *C. difficile* to the intestinal wall [7]. The S-layer proteins (SLPs) of *C. difficile,* composed of a high molecular weight protein (HMW) and a low molecular weight protein (LMW), are potential colonization factors thought to be involved in bacteria-host interactions [8], [9], [10]. O’Brien *et al* tested *in vivo* the efficacy of anti-SLP to prevent CDI: passive immunization using anti-SLP antibodies significantly delays the progress of CDI in the hamster model [11]. SLPs were also tested as vaccine component in hamsters but did not fully protect the animals, and antibody production was variable and generally modest or poor [12]. In a previous study, we showed that *C. difficile* cell wall extracts (CWE) used as antigens for intra-rectal immunizations were able to delay *C. difficile* colonization in a human microbiota-associated mouse model [13]. The aim of that study was to evaluate *C. difficile* s as vaccine candidates in the hamster model of CDI. We assessed the protective effect of immunization by following the kinetic of animal death after challenge with a toxigenic *C. difficile*. In addition, we studied the immune response of hamsters against a *C. difficile* CWE using a proteomic approach. After identification of proteins revealed by the immune-proteomic approach, the ability of one of these proteins, the heat shock protein GroEL, to induce protection against *C. difficile* colonization by immunization was in a conventional mouse model.

## Materials and Methods

### Ethics statement

The protocols involving animals and their care were conducted in conformity with the institutional guidelines that are in compliance with national and international laws and policies (Decree 87-848, october 19, 1987 modified by the decree 2001-464, may 29, 2001, Ministère de l'agriculture et de la pêche, permission # B92-019-01, Préfet des Hauts de Seine). All efforts were made to minimize animal suffering. The protocol was approved by the Committee on the Ethics of Animal Experiments of the University of Paris-Sud.

### *Clostridium difficile* strains

The *C. difficile* strain 79-685 is Tcd A and Tcd B positive. This strain was isolated in a patient with pseudomembranous colitis in France. We used this strain for animal challenge in order to develop *C. difficile* infection.

The *C. difficile* strain ATCC 43603 is non-toxinogenic (TcdA-, TcdB-, binary toxin negative), PCR-ribotype 085. This non-toxinogenic strain has been used for cell wall extracts immunization in order to avoid animal protection being related to the presence of antitoxin antibodies triggered by the toxins present in the cell wall extract preparations. Strains were grown as previously described [13].

### Preparation of cell wall extracts (CWE) and recombinant GroEL

Surface proteins of *C. difficile* strain ATCC 43603 were extracted as described by Wexler *et al.* [14]. The bacteria were cultured at the stationary phase in 100 ml of tryptone glucose yeast medium under anaerobic conditions. After centrifugation the pellet was washed 3 times with PBS, and suspended in a 0.062 M Tris buffer pH 6.8. Glass beads (Sigma) diameter 0.1 mm were added. The mixture was then vortexed and centrifuged. The supernatant containing the extrated proteins was aliquoted and stored at −20°C until used. Recombinant GroEL was purified as previously described [15].

### Preparation of spores

Spores of the *C. difficile* strain 79-685 were prepared as previously described [16].

### Animals

Two animal models have been used: the hamster model, which allows to observe animal protection against infection but that is not the most suitable to follow protection against the *C. difficile* colonization. The mouse model is the conventional model to monitor *C. difficile* intestinal colonization [17].

**Hamster model of protection.** Adult *Mesocricetus auratus* female hamsters (weight, 80–100 g), obtained from Elevage Janvier (France), were housed individually in micro-isolator cages. All food, water, bedding and cages were autoclaved before being used.

*In vivo* experiments were performed at the animal central care facility of the faculty of Pharmacie, University Paris-Sud. After infection of animals by *C. difficile* 79-685 spores, hamsters may develop diarrhea characterized by a wet tail and traces of liquid stool in the litter, mild, moderate or severe weight loss, and decrease in activity. Once symptomatic, hamsters were observed at 8-h intervals and based on signs of diarrhea and weight loss, humanely euthanized if necessary. Hamsters were humanely euthanized when they had a weight loss greater than 10% of their initial weight, and / or a significant decrease in activity. In the case of absence of clinical signs, the animals were humanely euthanized at the end of the study. Hamsters were humanely euthanized using a lethal dose of pentobarbital (150 mg / Kg) intraperitoneally. Hamsters were anaesthetized during collections of blood and during intra-rectal immunizations by intraperitoneal injection of Ketamine ® 1000 (100 mg / Kg), Rompun ® 2% (0.25 mL / Kg).

**Mouse model of colonization.** Six to eight week old female C3H mice were obtained from Elevage Janvier (France). Mice were caged in groups of five. All food, water, bedding and cages were autoclaved before being used. In this mouse model, animals do not develop clinical manifestations of infection. Mice were anaesthetized during the collection of blood and during intra-rectal immunizations by intraperitoneal injection of 150 µL of a cocktail composed of 30 µL of Imalgene® 1000, 15 µL of atropine sulfate 1 mg/mL and 9.5 µL of valium® 10 mg / mL. Mice were sacrificed at the end of the experiment by cervical dislocation.

### Immunization regimen

Hamsters and mice are different animal models for *Clostridium difficile* infection. For the mouse model, several mucosal route of immunization are possible. We selected the nasal route since it is clearly more practical and allowed for the production of specific serum antibodies.

**Hamster model.** Immunization trials were performed in duplicate on hamsters using *C. difficile* cell wall extracts (CWE). The total animal number was 16 and 20 respectively for the control group and for the CWE immunized group. The immunization assay has been done with six control hamsters and seven hamsters immunized by the recombinant GroEL protein.

Hamsters were intra-rectally immunized with either 10 µg of Cholera toxin (CT) (Servibio laboratories - France) for the control group or a combination of 10 µg of CT and 300 µg of CWE or 100 µg of GroEL respectively for the assay groups. Hamsters were immunized on days 0, 15 and 30. Prior to immunization, hamsters were anaesthetized.

Fifteen days after the final immunization, hamsters were orally administered clindamycin (Dalacine®) with a single dose of 50 mg/Kg to disrupt the barrier microbiota. Five days after, hamsters were challenged by oral administration of 2×10^3^ spores of *C. difficile* 79-685.

**Mouse model.** The control group of six mice was immunized intra-nasally with 20 µl of phosphate-buffered saline (PBS) and 5 µg of cholera toxin. The test group of six mice received intra-nasally 12 µg of purified recombinant GroEL and 5 µg of cholera toxin. Mice were immunized with four doses on days 0, 7, 14, and 28. To disrupt the barrier microbiota, mice were orally administered Cefoxitin (Mefoxin®) for five consecutive days, with a dose of 100 mg/Kg, fifteen days after the last immunization. A day later, mice were challenged by oral administration of 10^9^ cells of *C. difficile* 79-685.

### Serum sampling for immune analysis

To evaluate serum antibody response, blood samples were withdrawn before the first immunization and 15 days after the last immunization, before *C. difficile* challenge. Each hamster was sampled under an anaesthetic directly by heart puncture, and each mouse was sampled from the retro-orbital sinus.

### *C. difficile* detection in hamster and mouse fecal samples

Fecal pellets were cultured before antibiotic administration and daily for one week after *C. difficile* challenge, to assess the colonization rate. Ten mg of feces were suspended in one mL of LCY, and 100 µL of ten-fold serial dilutions were cultured in anaerobiosis on Columbia agar containing 5% of horse blood, 25% (w/v) of D-cycloserine, and 0.8% (w/v) of cefoxitin. Typical fluorescent colonies were counted under UV light (312 nm). By using this method, the threshold of *C. difficile* detection in animal feces was 10^4 ^CFU/g.

### Two-dimensional gel electrophoresis (2-DE) of *C. difficile* cell wall extracts

Prior to 2-DE, surface protein extracts were precipitated with Proteo-Extract (Calbiochem, EMD Biosciences) following the manufacturer’s instructions and the pellets were solubilized in rehydratation buffer (7 M urea, 2 M thiourea, 3% w/v CHAPS, 1% v/v Nonidet P-40, 0.5% v/v IPG buffer, 1% w/v DTT, bromophenol blue). 2-DE were performed in duplicate using large gels (200×250×1 mm) and small gels (70×90×1 mm). In each experiment, 3 gels were realized. A total of 200 µg of proteins was loaded on 18 cm, pH 4-7 linear immobilin dry strips (IPG strips, GE Healthcare Biosciences-AB) and 80 µg on 7 cm, pH 4-7 linear IPG strips for isoelectrofocusing step (IEF). Two-dimensional gel electrophoresis (2-DE) was performed as previously described [18]. Two gels were used for immunoblotting; the third gel was fixed and stained with Coomassie Brilliant Blue (Sigma).

### Immunoblotting

Cell wall extract proteins separated by 2-DE were transferred onto PVDF membranes (Amersham biosciences) as previously described [19]. One membrane was incubated for 2 h with pooled CWE-immunized hamster sera diluted at 1∶3,000; the other was incubated with the pool sera of control hamsters. We defined the proteins of interest as those revealed by cell wall-immunized hamster sera, but not by control hamster sera.

### Protein identification

Spots of interest were manually excised in-gel digested according to a standard trypsin protocol. The tryptic fragments were extracted in 60% ACN and 5% formic acid and analyzed by MALDI-TOF MS and by nanoLC-MS/MS [18]. The MS and MS/MS spectra were matched automatically to proteins in NCBI database with 5470121 sequences and 1894087724 residues using the Mascot search engine (v2.2) Peptide Mass Fingerprinting and MS/MS Ion Search algorithms (http://www.matrixscience.com/). Protein identification was only accepted with a significant molecular weight search (MOWSE) score (p value < 0.05).

### Detection of GroEL specific antibodies in sera of CWE immunized hamsters and GroEL immunized mice

ELISA was used to detect antibodies in the sera as described before [13]. Sample dilution tested was 1∶100 for hamster and 1∶2 for mice. Positive reactions were detected with i) for hamsters: rabbit anti-hamster immunoglobulins conjugated to biotin (1∶8,000 dilution; Biovalley) and ii) for mice: goat anti-mouse IgG conjugated to biotin (1∶20,000 dilution; Sigma).

### Statistical analysis

The survival of animals following infection was analyzed using Kaplan-Meier estimates. Survival rates across groups were compared using log rank tests. p values < 0.05 were considered to be statistically significant. Statistical analyses were performed using Stata 8.0 (Statacorp, College Station, TX).

For antibody response, as antibody levels were not normally distributed, we used Wilcoxon’s rank score test to test the null hypothesis that there was no difference between the immunized group and the control group. Analyses were done with the Stata 8.0 (Statacorp, College Station, TX). Statistical significance was set at p  =  0.05. All p values were one-sided.

For the mice immunization assay, statistics were done separately on day 1, 4, 8, 10 and 14. As CFU of *C. difficile* per g of fecal sample were not normally distributed, we used Mann–Whitney U test for non-parametric data to test the null hypothesis that there was no difference between the immunized group and the control group.

## Results

### Preparation of cell wall extracts

The strains 79-685 and ATCC 43603 are similar in terms of the surface proteins as shown by SDS-PAGE realized with their cell wall extracts (Fig. S1). In addition, specific antibodies against the known surface proteins (Cwp66, Fbp68, GroEL, FliC, FliD and Cwp84) recognized the corresponding proteins in the cell wall extracts of the two strains by immunoblot (data not shown).

### Hamster immunization with *C. difficile* CWE

We conducted experiments to test the ability of cell wall proteins (Fig. S1) to protect hamsters from a primary challenge with *C. difficile*. All animals were predisposed to *C. difficile* infection by clindamycin treatment. Hamsters were then challenged with *C. difficile* spores twenty days after the last immunization, and their survival was monitored.

**Figure 1:**
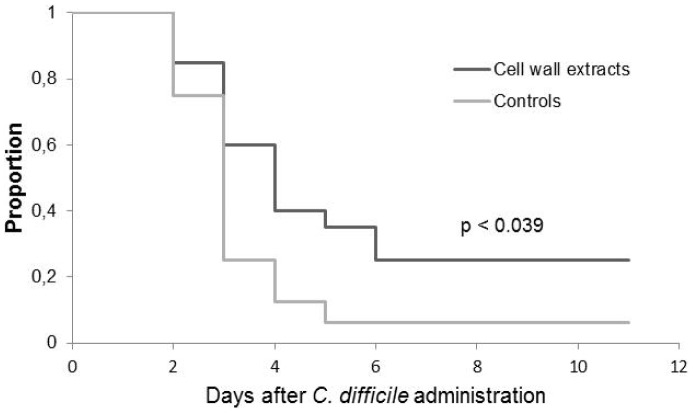
Kaplan-Meier survival estimates demonstrating time between challenge with *Clostridium difficile* and death. Clindamycin (50 mg/Kg) was administered 5 days before spore challenge. Animals were observed for 11 days. Experiments were performed with hamsters receiving PBS as control (n = 16) and hamsters immunized intra-rectally with cell wall extracts (CWE) (n = 20). Cholera toxin was used as adjuvant, for the two groups.

**Figure 2:**
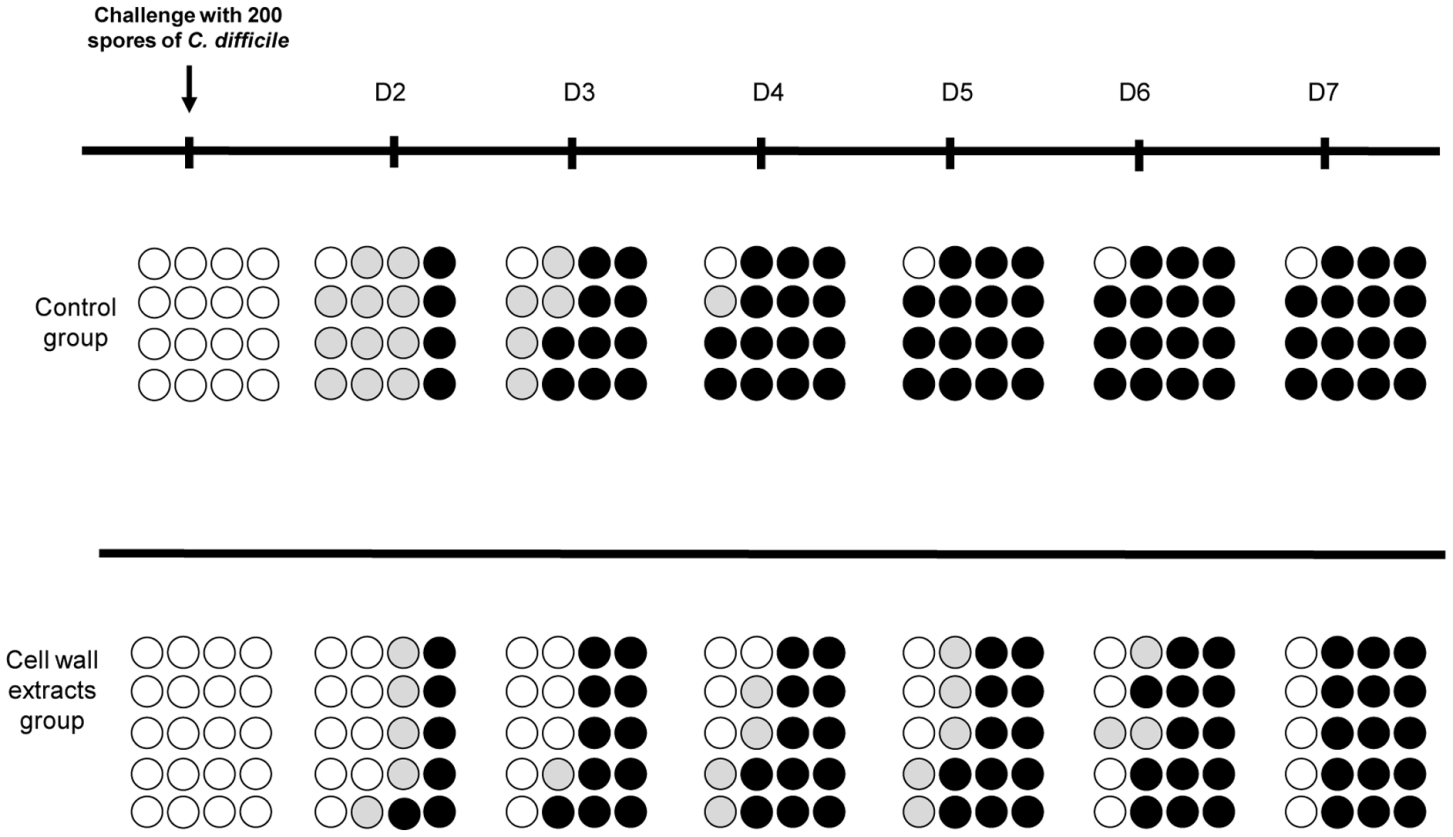
Course of colonization and death of hamsters challenged with *Clostridium difficile* after pre-treatment with clindamycin. Each circle represents the same animal on different days (D) of observation. White circles: uncolonized hamsters; grey circles: colonized hamsters, black circles: colonized hamsters that died.

Hamsters immunized with the CWE by the rectal route had a longer survival with a statistical significant difference compared to the control group (p  =  0.039) (Fig. 1). All animals that died were colonized by *C. difficile*. In the control group, colonized hamsters demonstrated 100% mortality, the only one that survived was not colonized by *C. difficile*. The survival of hamsters mirrored the level of *C. difficile* recovered in hamster fecal pellets. The percentage of hamsters colonized differed according to the groups. On day 2, the 79-685 *C. difficile* strain colonized 94% (15/16) of hamsters in the control group whereas only 55% (11/20) in the immunized group (Fig. 2).

CWE-immunized hamsters were less colonized by *C. difficile* than hamsters from the control group. On day 4, three hamsters of the immunized group were weakly colonized with less than 10^6^ CFU/g of feces. *C. difficile* was no longer detectable two days later and these hamsters finally survived to the challenge. Moreover, the group immunized with CWE was later colonized by *C. difficile*. In the immunized group, intestinal colonization progressed for seven days after the challenge, while colonization of the control group occurred within the first 2 days.

Signs of morbidity such as inactivity and wet tail or diarrhea were not always apparent before death.

### Identification of immunoreactive proteins

**Figure 3:**
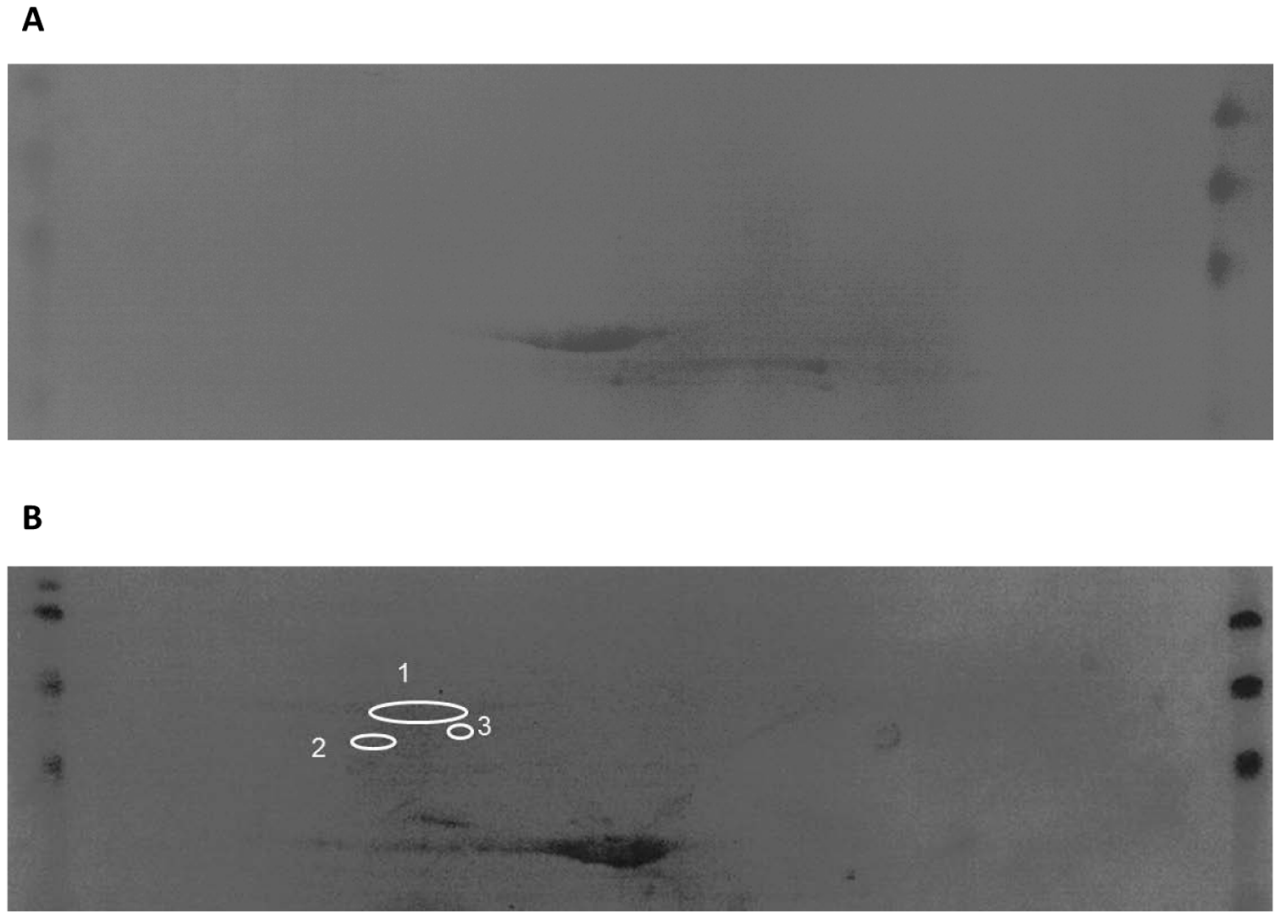
Immunoblots of cell wall extracts revealed by sera of hamster from the control group (A) and cell wall extracts revealed by sera of hamster from the cell wall extracts immunized group (B). Spots circled correspond to immuno-reactive proteins, DnaK (1), GroEL (2), S-layer protein precursor (3).

In order to identify the *C. difficile* surface proteins able to induce the immune protective response in hamsters, an immuno-proteomic approach was taken. The cell wall protein extract used for immunization was separated by 2-DE. Triplicate gels were prepared, for two of them the proteins were transferred to a PVDF membrane. Figure 3 shows one membrane probed with a pool of hamster-control anti-sera (Fig.3A), and the other, with hamster-immunized anti-sera (Fig.3B).

The comparison between the two membranes revealed respectively by the control and the immunized anti-sera allowed detecting differential immuno-reactive proteins.

**Figure 4:**
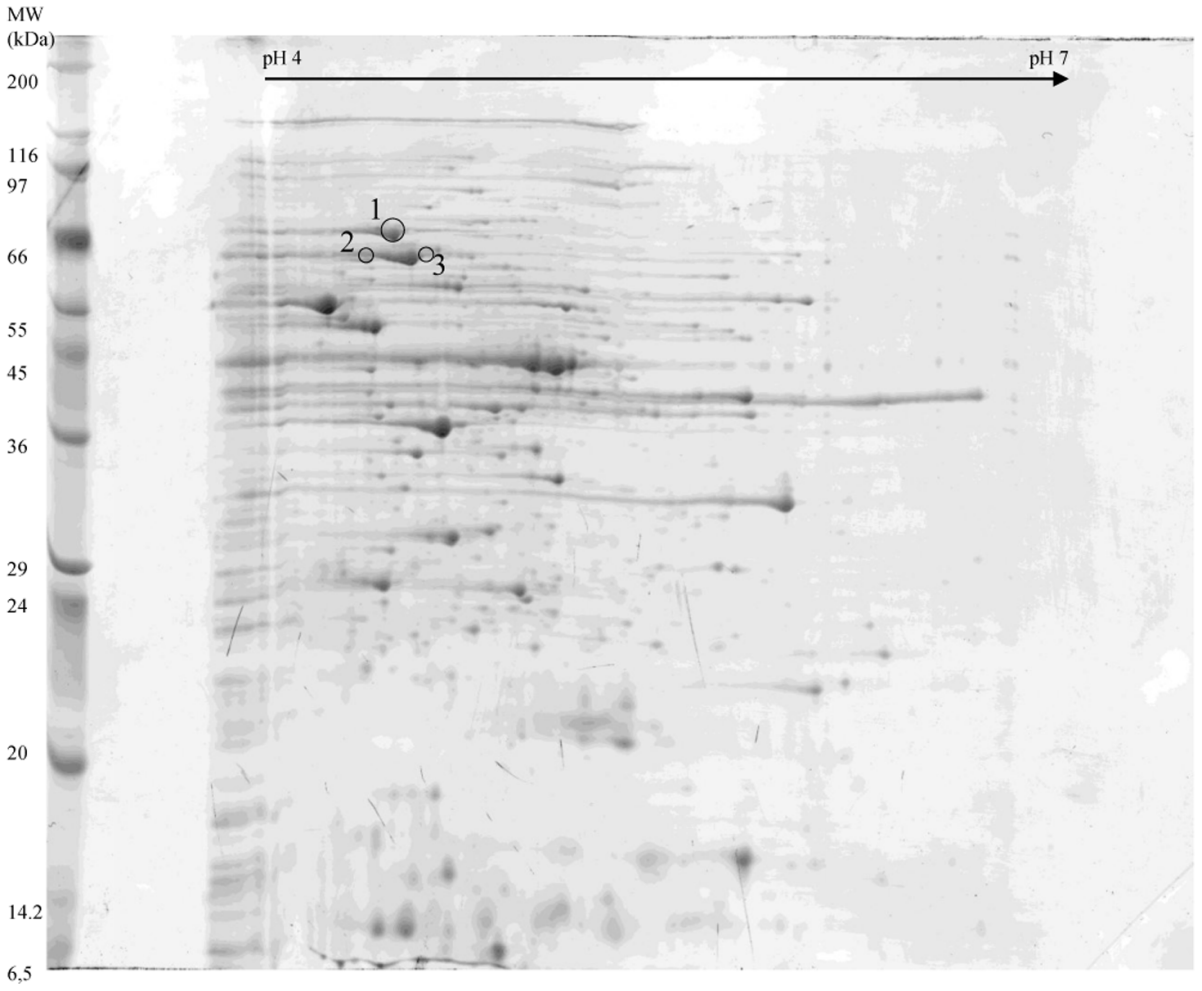
2-DE map on 18 cm IPG strip of cell wall proteins extracts used for hamsters immunization. Spots circled correspond to immuno-reactive proteins, DnaK (1), GroEL (2), S-layer protein precursor (3).

Immunoblots were compared to the 2-DE gel stained with brilliant blue (Fig. 4). Spots circled are those that were immuno-reactive with hamster-immunized sera but not with hamster-control sera. These immuno-reactive proteins were picked from the stained gel to determine their identity. A total of six spots were identified and proven, revealing the identity of three different proteins: a heat shock protein (Hsp) GroEL, another chaperon protein identified as Hsp 70 (or DnaK) and the S-layer protein precursor (Table 1).

**Table 1:** Protein identification by MS and MS/MS analysis.

Spot ID	Protein identification	Theoretical Molecular mass (Da)	Theoretical pI	Number of matching peptides	Sequence coverage (%)	MOWSE score
1	Chaperon protein DnaK	66541	4.72	13	20	661
2	Heat shock protein GroEL	57684	4.75	15	27	122
3	S-layer protein precursor	76101	4.78	13	23	748

### Detection of GroEL specific antibodies in sera of CWE immunized hamsters

**Figure 5:**
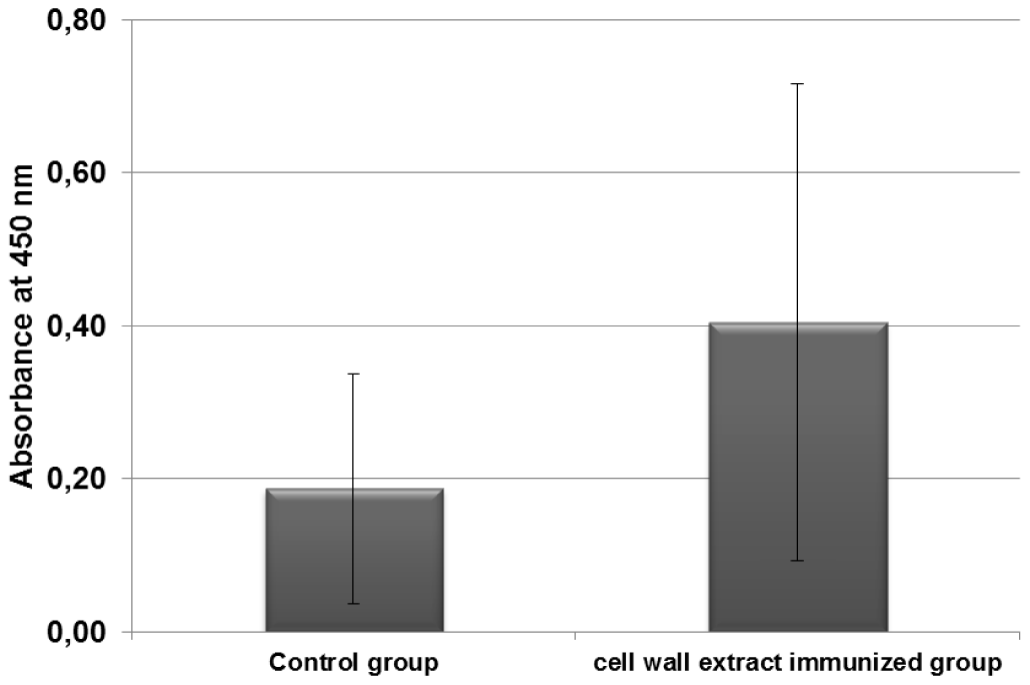
GroEL-specific antibodies in serum of the CWE-immunized hamster group and in the control group. Sera of hamster (diluted 1∶100) were analyzed by ELISA.

Hamsters have been immunized by CWE, which is a pool of surface proteins. Among these proteins, three appeared to be immuno-reactive, including GroEL. To confirm that GroEL-specific antibodies could be implicated in the hamster protection after *C. difficile* challenge, we first confirmed the presence of GroEL in the cell wall extracts by immunoblot, using specific GroEL antibodies (Fig. S2). Secondly, we searched for GroEL-specific antibodies in hamster sera by ELISA. We compared the GroEL-specific antibody level in the CWE-immunized group and the control group. The mean of absorbances at 450 nm was significantly higher for the CWE-immunized group (0.561) than for the control group (0.344) (p =  0.03) (Fig.5).

### Immunization with recombinant GroEL

GroEL has been shown to be putatively involved in the colonization process of *C. difficile*. As partial protection of hamster after CWE immunization could be related, at least partially, to host production of antibodies, we have attempted to assess the ability of GroEL immunization to induce a protection against *C. difficile*. We therefore performed an immunization with GroEL in the hamster model. The immunization assay was performed with six control hamsters and seven hamsters immunized with the recombinant GroEL protein. However, hamsters immunized with GroEL by the rectal route did not show a longer survival compared to the control group (p  =  0.736). These results suggest that GroEL immunization may lead to partial inhibition of colonization, but this effect was not strong enough to reduce disease burden in hamsters. The decrease of bacterial colonization was observed in our mouse model. The speed at which the decrease occurred could not be fast enough to limit toxins A and B action in the hamster model.

Hamsters and mice are different animal models for *Clostridium difficile* infection. For the mouse model, several mucosal route of immunization are possible. We selected the nasal route which allowed the production of specific seric antibodies and which is more practicable.

Two groups of six animals were immunized by intra-nasal route. A control group was immunized with 5 µg of cholera toxin diluted in PBS, while the other group was immunized with 5 µg of cholera toxin added to 12 µg of recombinant GroEL protein. Twenty days after the last immunization, after disruption of the barrier microbiota with an antibiotic treatment, the mice were challenged with the virulent *C. difficile* strain 79-685. *C. difficile* colonization was monitored by numeration of *C. difficile* in animal fecal samples after challenge.

**Figure 6:**
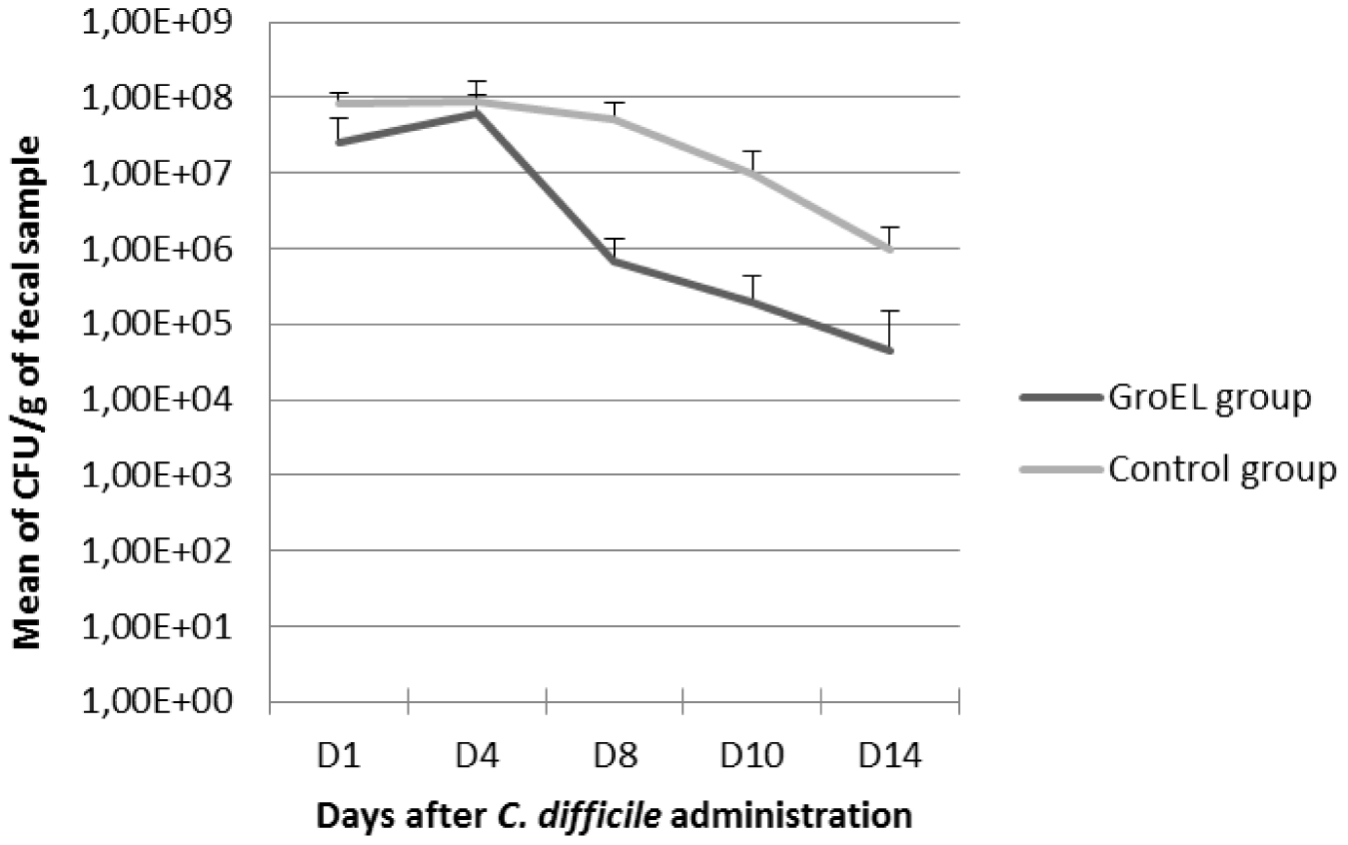
Evaluation of intestinal colonization of mice by *C. difficile*. Colonization was evaluated at days 1, 4, 8, 10 and 14 in the GroEL- immunized group and in the control group after intra-gastric administration of *C.difficile* to mice.

As shown in Fig. 6, from day 4, the *C. difficile* fecal bacterial count was greater in the control group than in the GroEL immunized group, and significantly at day 8 (p = 0.0079) and day 10 (p = 0.0079). Thus, immunization with GroEL led to a decreased colonization of the host in the mouse model.

**Figure 7:**
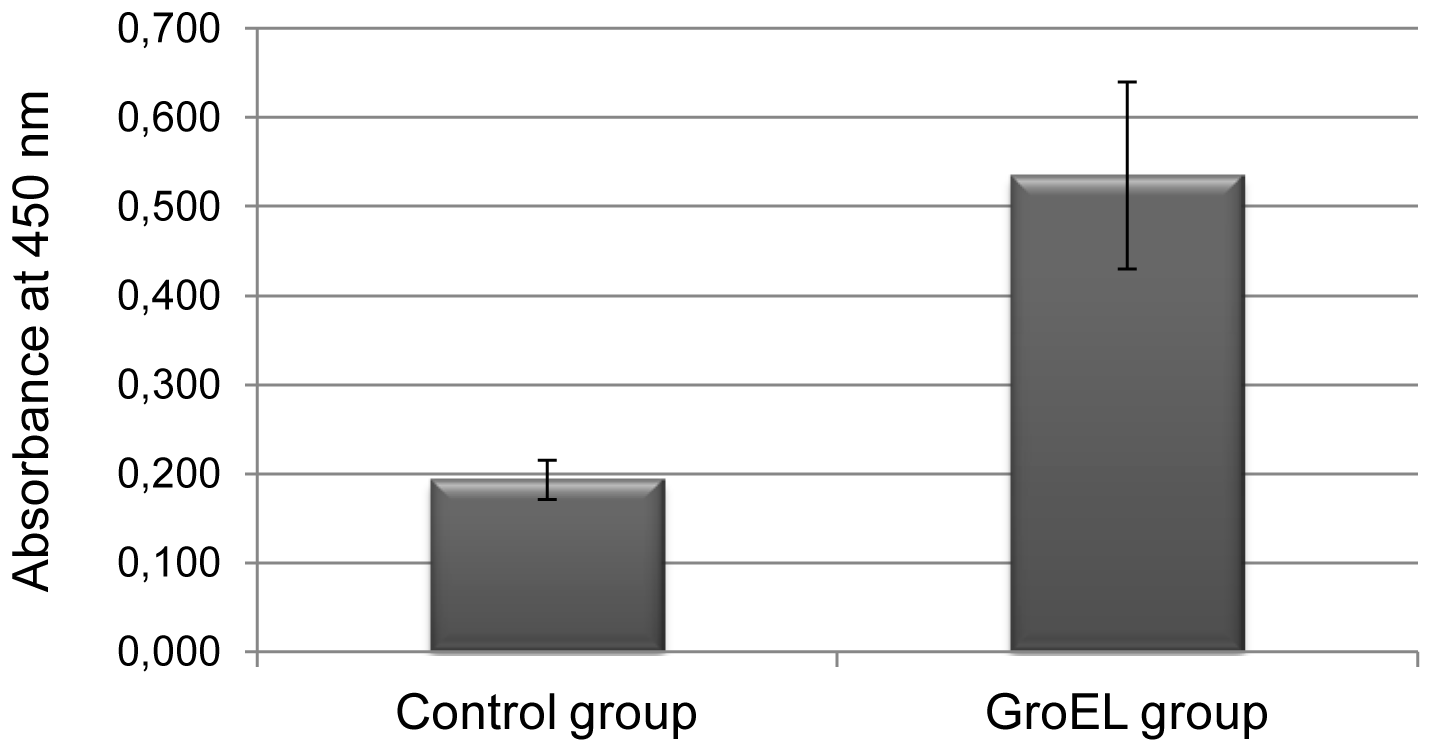
GroEL-specific antibodies in the serum of mice. Sera (diluted 1∶2) of GroEl immunized mice group and of the control group, immunized by the intranasal route, were analyzed by ELISA.

The lower level of *C. difficile* intestinal colonization in the GroEL immunized group can be related to the immune response. In fact, as shown in Fig. 7, detection of specific GroEL antibodies in mice sera revealed that the level was higher in the immunized group compared to the control group with a statistically significant difference (p =  0.003).

## Discussion

Toxins have been candidates for a *C. difficile* vaccine. However, since toxins are released molecules, it is not clear that an effective antibody response directed to toxins will eliminate carriage of *C. difficile*.

The surface exposed antigens have been long emphasized as important vaccine candidates since they are involved in the first line of bacterium-host interactions.

In a previous study, we demonstrated that an intra-rectal immunization of hamsters with the surface-associated protease Cwp84 resulted in a weaker and slower *C. difficile* intestinal colonisation. Furthermore, the hamster survival in the Cwp84 immunised group was greater than that of the control group with a significant statistical difference [20]. But, as the colonization phase involves several factors, is may be necessary to associate several surface proteins of *C. difficile* in order to totally protect animal from death.

We previously demonstrated the ability of an intra-rectal immunization with CWE for reducing *C. difficile* intestinal colonization in a human microbiota-associated mouse model [13]. Here we used the same immunization regimen in the hamster model which is highly susceptible to CDI. So, we tested the animal death protection after CWE immunizations. We confirmed in the hamster model the decrease of *C. difficile* intestinal colonization, associated with a protection of 25% of the hamsters against death with a statistically significant difference compared to the control group. This protection could be due to an immune response directed to proteins involved in the colonization process.

Our approach in the immuno-reactive protein identification was to examine the hamster immune response to cell wall surface proteins, highlighting the proteins involved in the hamster colonization process, and putatively, in the hamster protection. The proteomic analysis revealed three different proteins: two heat shock proteins GroEL and DnaK (Hsp 70), and the precursor of the S-layer proteins.

Being able to identify the S-layer precursor is not very surprising. Indeed, SLPs are abundant surface proteins of *C. difficile*. However, vaccine experiments with extracted SLPs used by different routes led to a partial protection of animals against CDI [12].

As a novel vaccination approach, heat shock protein-based vaccines have become an attractive strategy for disease prevention. HSP are usually intracellular proteins highly conserved and abundant proteins produced by all living cells in response to a variety of physiological insults and ensure survival under stressful conditions. However, when they act as danger signals during infections, they need to be present in the extracellular environment. Many bacterial HSPs have been found in the extracellular medium and implicated in immune reaction to infections [15], [21], [22]. Microbial HSPs have been reported to be dominant antigens for the host immune response to a variety of pathogens, and the immune recognition of these HSPs serves as a first line of defense [23], [24]. Growing evidence that extracellular HSPs function as endogenous immuno-modulators for innate and adaptive immune response have been demonstrated. Recently, a number of studies reported significant protection by using HSPs as vaccines in various infection disease model [25], [26]. Microbial HSPs have been suspected to be involved in auto-immune diseases due to molecular mimicry of conserved sequences between host and pathogen [27], [28]. However, recent studies have shown that rather than promoting disease, cross-reactivity can suppress auto-immunity through regulatory function of HSP-reactive T-cells and IL-10 production [29], [30] as seen in case of adjuvant-induced arthritis [31] and diabetes [32] after HSP vaccination in animal models. HSPs can, therefore, be safely used as vaccines. Since heat shock proteins have partial human homology, however, future work could be done to identify possible protective portions or epitopes of this antigen lacking the human homology.

Intraperitoneal immunizations of mice with GroEL of *Streptococcus pneumoniae* delay death [33]. Regarding *Salmonella enterica* serovar Typhi, immunization of mice with recombinant GroEL protein conferred 70-90% protection against lethal infections either by *S.* Typhi Ty2 or *S. Typhimurium* [34].

Recently, GroEL and DnaK of *Bacillus anthracis* were investigated for their immunogenicity and protective efficacy. Immunization with GroEL conferred 100% protection to mice against *B. anthracis* infection whereas DnaK could not provide protection [35]. This led us to select GroEL as antigen among the three proteins identified by proteomic. Hamster protection observed after CWE immunization is not the result of only one antigen. Intestinal colonization is a very complex process involving several colonization factors. Therefore, the use of a single surface protein as a vaccine antigen had a minor impact on hamster protection against *C. difficile* infection. For this reason, we chose to perform a second immunization assay in a mouse model, which is more suitable to analyses the impact of immunization on the colonization process.

GroEL of *C. difficile* is released extra-cellularly after heat shock, but can also be surface-associated. [15]. The decrease of *C. difficile* intestinal colonization observed in mice after GroEL immunization correlated with a high specific antibody response, confirmed previous results suggesting a role for GroEL in the adhesion process [15].

Mucosal surfaces are the primary sites for transmission of most infectious diseases. Rectal vaccination has been previously tested against enteric pathogens such as *Salmonella* [36], [37]. As preliminary assay, our work has the merit to indicate the efficacy of a vaccine targeting the colonic mucosa. We previously, succeeded in targeting the colonic mucosal site by an oral route of immunization by antigen encapsulation [16].

In this study, after CWE immunization in a hamster model, we induced a decrease of *C. difficile* intestinal colonization and a significant survival. We showed that the immune response of hamsters is directed against several major surface proteins including the SLPs and HSPs including GroEL. The specific role of GroEL on the colonization process has been confirmed by an immunization assay in a colonization mouse model. These results highlight the interest of a combination of surface proteins as vaccine antigens in order to prevent *C. difficile* intestinal colonization and infection development.

## Supporting Information

Figure S1 **SDS-PAGE analysis of cell wall extracts from ATCC 43603** ***C. difficile*** **strain (1) and 79-685** ***C. difficile*** **strain (2).**
(TIF)

Figure S2 **Immunoblot of cell wall extracts from the ATCC 43603** ***C. difficile*** **strain revealed by GroEL specific antibodies (1).**
(TIF)
