# Supplementary figures and images for: Immunization Using GroEL Decreases Clostridium difficile Intestinal Colonization
Source: PLoS One. 2013 Nov 26;8(11):e81112. doi: 10.1371/journal.pone.0081112 (PMC3841151; doi:10.1371/journal.pone.0081112)

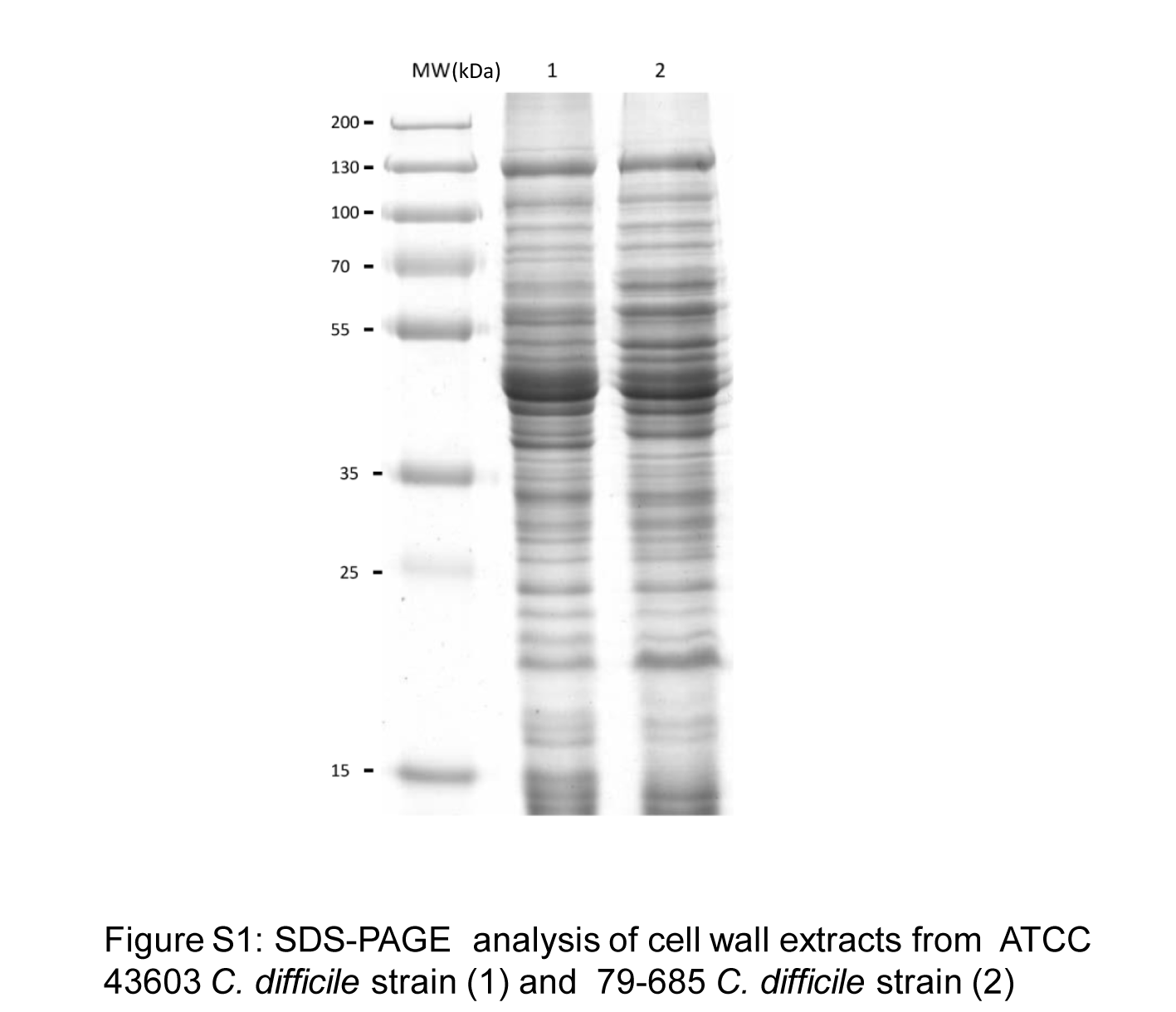

Supplement: Figure S1 — SDS-PAGE analysis of cell wall extracts from ATCC 43603 C. difficile strain (1) and 79-685 C. difficile strain (2). (TIF) [file pone.0081112.s001.tif]

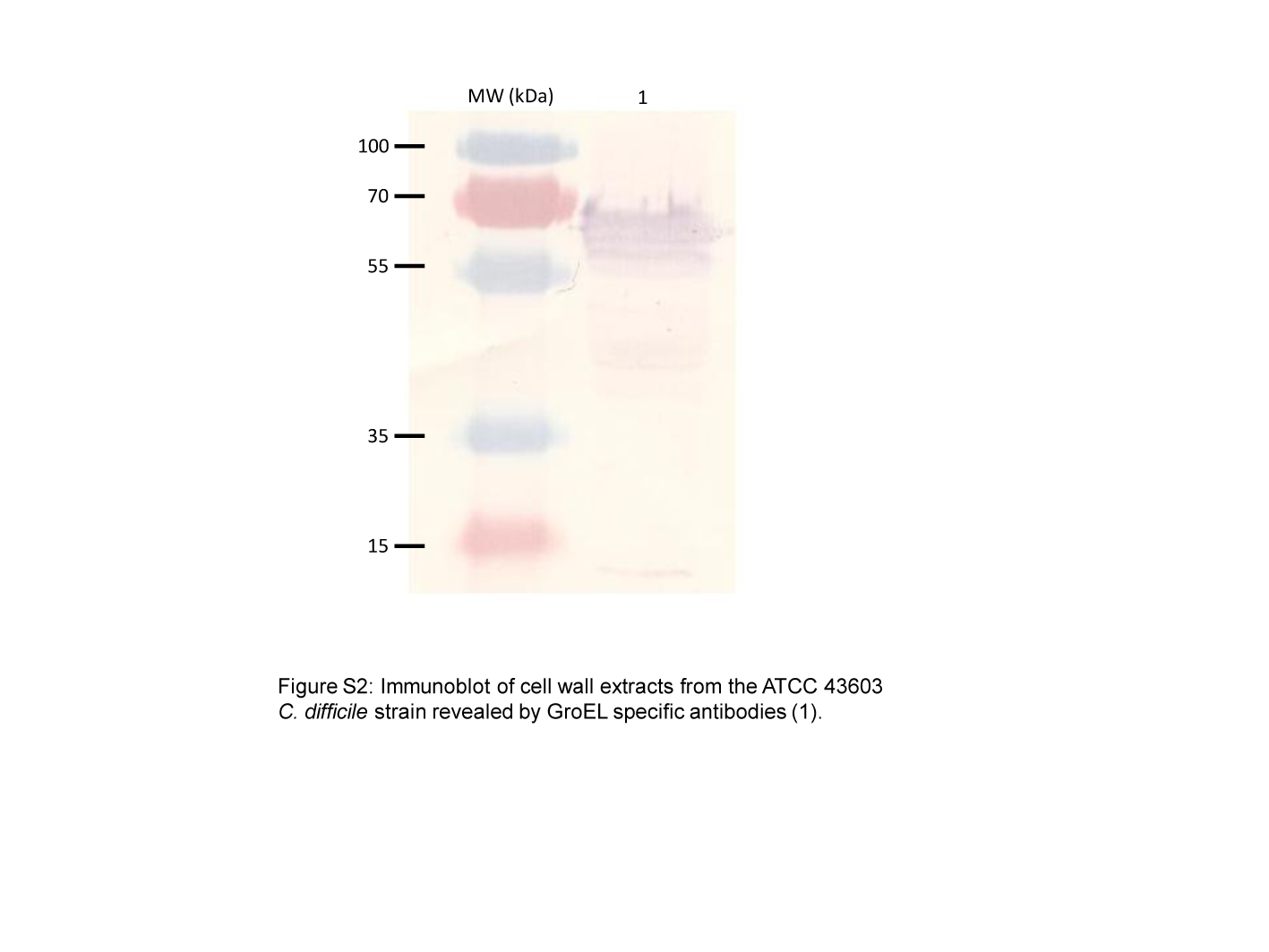

Supplement: Figure S2 — Immunoblot of cell wall extracts from the ATCC 43603 C. difficile strain revealed by GroEL specific antibodies (1). (TIF) [file pone.0081112.s002.tif]
